# Effects of naturally-produced lovastatin on feed digestibility, rumen fermentation, microbiota and methane emissions in goats over a 12-week treatment period

**DOI:** 10.1371/journal.pone.0199840

**Published:** 2018-07-05

**Authors:** Su Chui Len Candyrine, Mazrul Fahmi Mahadzir, Sani Garba, Mohammad Faseleh Jahromi, Mahdi Ebrahimi, Yong Meng Goh, Anjas Asmara Samsudin, Awis Qurni Sazili, Wei Li Chen, Siva Ganesh, Ron Ronimus, Stefan Muetzel, Juan Boo Liang

**Affiliations:** 1 Institute of Tropical Agriculture and Food Security, Universiti Putra Malaysia, Serdang, Selangor, Malaysia; 2 Faculty of Sustainable Agriculture, Universiti Malaysia Sabah, Sandakan, Sabah, Malaysia; 3 Agricultural Biotechnology Research Institute of Iran, Mashad, Iran; 4 Faculty of Veterinary Medicine, Universiti Putra Malaysia, Serdang, Selangor, Malaysia; 5 Faculty of Agriculture, Universiti Putra Malaysia, Serdang, Selangor, Malaysia; 6 Rumen Microbiology, AgResearch, Palmerston North, New Zealand; Gaziosmanpasa University, TURKEY

## Abstract

Twenty male Saanen goats were randomly assigned to four levels of lovastatin supplementation and used to determine the optimal dosage and sustainability of naturally produced lovastatin from fermentation of palm kernel cake (PKC) with *Aspergillus terreus* on enteric methane (CH_4_) mitigation. The effects on ruminal microbiota, rumen fermentation, feed digestibility and health of animal were determined over three measuring periods (4-, 8- and 12-weeks) and the accumulation of lovastatin in tissues was determined at the end of the experiment. The diets contained 50% rice straw, 22.8% concentrates and 27.2% of various proportions of untreated or treated PKC to achieve the target daily intake level of 0 (Control), 2, 4 or 6 mg lovastatin/kg body weight (BW). Enteric CH_4_ emissions per dry matter intake (DMI), decreased significantly (P<0.05) and equivalent to 11% and 20.4%, respectively, for the 2 and 4 mg/kg BW groups as compared to the Control. No further decrease in CH_4_ emission thereafter with higher lovastatin supplementation. Lovastatin had no effect on feed digestibility and minor effect on rumen microbiota, and specifically did not reduce the populations of total methanogens and Methanobacteriales (responsible for CH_4_ production). Similarly, lovastatin had little effect on rumen fermentation characteristics except that the proportion of propionate increased, which led to a decreasing trend (P<0.08) in acetic: propionate ratio with increasing dosage of lovastatin. This suggests a shift in rumen fermentation pathway to favor propionate production which serves as H^+^ sink, partly explaining the observed CH_4_ reduction. No adverse physiological effects were noted in the animals except that treated PKC (containing lovastatin) was less palatable at the highest inclusion level. Lovastatin residues were detected in tissues of goats fed 6 mg lovastatin/kg BW at between 0.01 to 0.03 μg/g, which are very low.

## Introduction

Mitigation of ruminant methane (CH_4_) emissions helps protect the environment as enteric CH_4_ emissions account for approximately 17% of the total global anthropogenic CH_4_ production [1]. Many nutritional CH_4_ mitigation approaches such as feed supplemented with essential oils [2], nisin [3] and condensed tannins [4] have been reported, but they are unlikely to be accepted at the farm level because the effects are often temporary as rumen microbiota can adapt to changes in the rumen ecosystem created by the mitigating agents. In addition, some CH_4_ mitigating agents also inhibit growth of useful rumen microorganisms such as peptidase producing and cellulolytic bacteria [5–7] leading to lower fiber digestion in the rumen. More recently, enzyme- and gene-based approaches have been studied [8]. Lovastatin, an inhibitor of 3-hydroxy-3-methyl-glutaryl coenzyme A reductase (HMG-CoA reductase), has been reported to effectively inhibit the HMG-CoA reductase activity which is necessary for biosynthesis of the cell membranes of archaea and thus selectively suppressing growth of methanogens without affecting other microorganisms in the rumen microbial community [9–10]. However, the above studies have made use of commercial lovastatin [9], a drug designed for lowering blood cholesterol in humans, which is too expensive to apply in longer-term animal studies. In addition, one study [10] was an *in vitro* study.

The efficacy of red yeast rice, a traditional Chinese health food supplement produced by fermentation of normal rice with *Monascus purpureus* (containing lovastatin in the form of monacolin K) on CH_4_ mitigation has recently been reported in cattle [11] and goats [12]. Although statistically significant, the reduction in g CH_4_ emission per kg DM intake of goats fed with red yeast rice (4.3 mg lovastatin/kg body weight (BW) was only 13% compared to the control animals. While in the case of cattle, the effect on CH_4_ emission was mild but severe adverse effects on DM intake, and clinical manifestation of digestive, muscular and urinary system disorders were reported when the animals were fed with 2.6 mg lovastatin/kg BW per day for only four days [11]. We have reported that lovastatin, produced naturally using rice straw incubated with *Aspergillus terreus* (130 mg lovastatin in 500 mg rice straw in 30 mL buffered rumen solution) reduced *in vitro* CH_4_ production by 27% [10] and similar rate of reduction in CH_4_ emissions was obtained in goats fed with the treated rice straw at 4 mg lovastatin/kg BW [13]. Results of the above studies and that of Klevenhusen et al. [14] using sheep have suggested that the effective dosage of lovastatin to mitigate enteric CH_4_ emissions is approximately 4 mg/kg BW for sheep and goats, but lower for cattle. To date, all published *in vivo* studies on using lovastatin to mitigate CH_4_ emissions in ruminants have been of short-term duration (ranging from several days to about four weeks). Such short-term studies did not fully take into account the potential of the rumen microbiota adapting to the effect of lovastatin over time as well as the possible negative effects of long-term consumption of lovastatin on the health and wellbeing of the animals. Thus, the present study was designed to examine the responses including feed intake and digestibility, rumen fermentation, microbiota, and CH_4_ emissions, to dietary supplementation of increasing dosages of naturally produced lovastatin, over a 12-week period using goats as the animal model. Palm kernel cake (PKC), a readily available agro-byproduct, which is a common ingredient in ruminant feed, was used as a substrate incubated with *Aspergillus terreus*, to produce the required lovastatin.

## Material and methods

### Preparation of fermented palm kernel cake

Palm kernel cake (a byproduct from the extraction of palm kernel oil) used for this study was purchased from a local palm kernel oil mill from the state of Selangor, Malaysia. Fermented PKC was prepared using a solid state fermentation (SSF) procedure as reported by Jahromi et al. [15] with some modifications. The primary modifications to the fermentation protocol were (i) PKC (instead of rice straw) was used as the substrate, (ii) the quantity of substrate was increased from 200 g of rice straw in 2 L Erlenmeyer flasks (incubated at 25 °C in laboratory incubator) to 4 kg PKC (incubated in trays stacked on racks in a room installed with air-conditioners to control the ambient temperature from 22 to 25 °C) and (iii) initial moisture content was increased from 50 to 55%. Other incubation factors including inoculum (*Aspergillus terreus* ATCC74135) and pH (7.0) remained the same as previously reported. The PKC was incubated for 10 days and the harvested PKC were dried in the forced-air oven at 60 °C, ground through a 2 mm sieve and stored for later used. Samples of the fermented PKC were analyzed for total lovastatin and the proportions of the bioactive hydroxyacid and less active lactone forms using HPLC [15]. The treated PKC contained an average of 850 mg lovastatin/kg dry matter (DM).

### Experimental design and animal management

The experimental protocol was approved by the Animal Care and Ethics Committee of the Universiti Putra Malaysia (UPM/IACUC/AUP-R0087/2015). Twenty intact Saanen male goats (4–5 months old, average BW of 26 ± 3.4 kg) were randomly assigned in equal number to four treatments [0 (Control), 2 (Low), 4 (Medium) or 6 (High) mg lovastatin/kg BW to determine the effect of increasing dietary supplementation of lovastatin on feed intake and apparent total-tract digestibility, rumen fermentation, microbiota, and CH_4_ emissions in goats. The goats were housed in individual pens with free access to clear drinking water and mineral blocks. After two weeks of adaptation, during the first (4-week) measurement period goats were individually offered a restricted feeding level of 650 g/day in two equal portions (08:00 and 20:00). Adoption of the restricted feeding level, determined as 90% of the lowest *ad libitum* intake of the goats during the two weeks adaptation period, was to ensure all goats consume the daily rations offered to them during the entire experiment. The total mixed ration containing 50% rice straw, 22.8% concentrates and 27.2% of various proportions of untreated or treated PKC (Table 1) to achieve the target daily intake level of 0 (Control), 2 (Low), 4 (Medium) or 6 (High) mg lovastatin/kg BW. For the second (8-week) and third (12-week) measurements, the feeding level was increased to 700 g/day while maintaining the same four targeted daily intake levels of lovastatin. The latter was achieved by maintaining the amount of treated PKC (carrier of lovastatin) and increasing the proportion of other ingredients accordingly.

**Table 1:** Composition of diets (% in total mixed ration).

	Dietary treatments			
Ingredients (%DM)	Control	Low	Medium	High
Rice straw	50.0	50.0	50.0	50.0
Corn	3.54	3.54	3.54	3.54
Soybean meal	16.31	16.31	16.31	16.31
Vitamin premix	0.5	0.5	0.5	0.5
Mineral premix	0.5	0.5	0.5	0.5
Limestone	1.0	1.0	1.0	1.0
Ammonium Chloride	1.0	1.0	1.0	1.0
Untreated PKC	27.2	18.4	9.7	0.9
Treated PKC	0	8.8	17.5	26.3
ME ^c^ (MJ/kg)	8.63	8.63	8.63	8.63

Control, Low, Medium and High represent 0, 2, 4 and 6 mg lovastatin/kg BW, respectively

^c^ = calculated value

The DM, organic matter (OM), crude protein, calcium and phosphorus of feed samples were determined according to AOAC [16], gross energy was determined by bomb calorimeter, while neutral detergent fiber (NDF) and acid detergent fiber (ADF) were determined according to Van Soest et al. [17] (Table 2).

**Table 2:** Chemical composition of diets.

Composition (% DM)	Rice straw	Concentrate in different treatments			
		Control	Low	Medium	High
Dry matter (%)	88.4	88.9	90.8	92.1	93.0
Organic matter	81.1	87.9	83.6	89.3	85.0
Crude protein	4.7	31.2	27.6	33.7	30.3
NDF	81.5	60.2	48.7	44.5	44.9
ADF	56.3	29.4	29.7	23.7	21.3
Calcium	0.25	1.20	1.32	1.39	1.80
Phosphorus	0.08	0.59	0.52	0.61	0.69
Gross energy (MJ/ kg)	16.80	16.60	16.72	15.56	14.99

Control, Low, Medium and High represent 0, 2, 4 and 6 mg lovastatin/kg BW, respectively

### Digestibility trial and methane gas measurement

The research facility for this study has five respiration chambers for the measurement of CH_4_ emissions. Methane emissions of all the 20 goats in the four treatment groups were measured over three periods; at 4, 8 and 12 weeks of the feeding trial. Each CH_4_ measurement consisted of 5 goats, randomly selected from each treatment groups, was carried out for two consecutive days, making up to a total of 8 days per feeding period (5 goats per two days) to complete the measurement for the 20 animals. Before the CH_4_ measurements, animals were transferred to metabolic crates for a five-day apparent total-tract digestibility trial using the total fecal collection procedure [18]. After the daily total fecal outputs were determined, 10% of the daily feces from each animal was sampled and stored and later pooled by animal at the end of the 5 days digestibility trial, dried in a forced-air oven at 60°C for 48 hours, and ground through a 2mm sieve before being subjected to further analysis.

After the digestibility trial, the goats were then randomly assigned to the respiration chambers a day before the CH_4_ measurement started. All goats had previously (during the adaptation period) adapted to the chambers prior to the gas measurements.

The five open circuit respiration chambers were constructed following the design implemented at AgResearch, New Zealand [19]. The interior volume of each chamber is approximately 2.0 m^3^ and made of a clear polycarbonate shell attached to a galvanized iron frame. The whole front and rear of the chambers are doors. When opened, the front door allows the operator free accessibility to the feed and water troughs which are attached to the crate holding the animal inside the chamber. The rear door allows for transferring of animal in and out of the chamber as well as removing and cleaning of urine and feces collected in a tray below the floor of the crate. The chamber has an exhaust pipe (top rear end of the chamber) drawing air into the chamber through a small inlet opening at the top front end of the chamber.

The chambers were arranged in a row, in a well-ventilated experimental room installed with air-conditioners to maintain the room temperature of between 22 and 25 °C. The recovery rate for each chamber was determined before each measurement period. Because of the small size of the goats, the outlet air flow rate was set at approx. 200 L/min (measured using an anemometer (SDL300 Extech Instruments, USA). Gas measurement started at 08:00 h and CH_4_ and carbon dioxide (CO_2_) concentrations of the outlet air were determined using a portable gas analyzer (Horiba PG-300, Japan) and the averaged values (average of 20 readings at one reading per three seconds) were recorded hourly for twelve hours per day (08:00–20:00) for two consecutive days. The two days measurements are to account for the daily variation in methane emission (if any) and an average daily methane emission was computed from the two days methane emission values. At the same time, temperature and ambient pressure in the chambers were also recorded using a data logger (SD700 Extech Instruments, USA).

For the first two periods (4- and 8-week), on the day after the completion of gas measurements, rumen content sample of the goats was collected four hours after the morning feeding by using an oral stomach tube. For the final measurement (12-week), rumen content sample was collected directly from the rumen of the goats when the animals were slaughtered. The rumen content from each animal was then gently squeezed through four layers of cheesecloth, separating the feed material of the rumen content, to obtain the rumen fluid [20]. The pH of the rumen fluids were immediately determined and the fluid was divided into two portions; one was stored at -80 °C pending DNA extraction for rumen microbial community analysis, and the other was preserved in 25% metaphosphoric acid (w:v) and stored at -20 °C for volatile fatty acids (VFA) analysis.

The health and wellbeing of the animals, particularly feed intake and manifestation of digestive disorders were monitored closely, by visual observations on a daily basis, throughout the experiment by the attending veterinarian involved in the study. At the end of the feeding trial (12 weeks), the goats were slaughtered in accordance to the halal (as prescribed by the Muslim law) standard procedures (MS 1500: 2009 of the Department of Standards Malaysia, 2009). Prior to slaughtering, approximately 10mL of blood samples were collected through the jugular vein from each animal for hematology and biochemistry analyses. Approximately 20g each of the muscle tissue (*Longissimus dorsi*) and organ samples (brain, liver and kidney tissues) were also collected. Samples were kept on ice during sampling and immediately transferred and stored in -20°C after sampling, until subsequent use for determination of lovastatin residues in the samples.

### Lovastatin residue study

For the determination of lovastatin residues in muscle and organ (kidney, liver, brain) tissues, 1 g of the samples were each ground to homogeneity with 5 mL of methanol. The extracts were then dried at 70 °C for 10 hours before re-suspension with 60% acetonitrile and centrifuged at 20,000 *g* for 20 min. The upper layer of the extract was analyzed for lovastatin residues using HPLC performed according to the method as described previously [15]. Since the concentration of lovastatin residues in all samples were below the detectable level (1 μg) by HPLC, one sample of each tissue from the highest lovastatin treatment group (6 mg/kg BW) were sent for further analysis using Trap Liquid Chromatography Tandem Mass Spectrometer (LCMS/MS) (AB Sciex 3200Q) (Toronto, Canada) coupled with Perkin Elmer Flexar FX15 Ultra High Performance Liquid Chromatography (UHPLC) system (Massachusetts, USA). The LCMS was equipped with Synergi Fusion RP column (Phenomenex, 100 mm x 2.0 mm x 4 μM). The mobile phases were water with 0.1% formic acid (mobile phase A) and acetonitrile with 0.1% formic acid (mobile phase B), with total run time of 8 minutes. Chromatography was achieved with Multiple Reaction Monitor (MRM) method. The gradient run program was set at 10% to 90% mobile phase B from 0.01 min to 4 min, and hold for 1 min, before going back to 10% mobile phase B and re-equilibrated for 3 min.

### Quantification of rumen volatile fatty acids

Rumen fluid samples collected as described previously were centrifuged at 10,000 x *g* for 20 min and the supernatants were collected for analysis. Acetic, propionic and butyric acids were determined using gas-liquid chromatography with using a Quadrex 007 Series (Quadrex Corporation, New Haven, CT 06525 USA) bonded phase fused silica capillary column (15 m, 0.32 mm ID, 0.25 μm film thickness) in an Agilent 7890A gas-liquid chromatograph (Agilent Technologies, Palo Alto, CA, USA) equipped with a flame ionization detector (FID). The temperature of the injector was maintained at 220 ^o^C while that of the detector was maintained at 230 ^o^C. The column temperature was first set at 70 ^o^C to 150 ^o^C and was programmed to increase at the rate of 7 ^o^C/min in order to achieve optimum separation of peaks. The standard used for VFA determinations was 4-methyl-N-valeric acid (Sigma, St. Louis, Mo., USA).

### Analysis of rumen microbial communities

#### Isolation of genomic DNA

The rumen fluid samples for microbial analyses were centrifuged at 10,000 x *g* for 10 min and the DNA extracted from the precipitate using a QIAamp DNA Stool Mini Kit (Qiagen Inc., Valencia, CA) according to manufacturer’s protocols.

#### Deep sequencing

To investigate the rumen microbial communities in a more comprehensive manner, the DNA extracted from each sample was sent to Macrogen Inc. (Korea) for library construction and sequencing on the MiSeq platform (Illumina). A deep sequence metagenome analysis was carried out using the 341F (CCTACGGGAGGCAGCAG) and 806R (GGACTACTAGGGTTTCTAAT) primers, which targets the V3 and V4 regions of the 16S rRNA gene of archaea and bacteria [21]. Sequences were processed, clustered and taxonomically classified using the QIIME software package (Quantitative Insights Into Microbial Ecology) [22]. First, paired-end reads were merged using FLASH (Fast Length Adjustment of Short reads [23]. Clustering and determination of OTUs (Operational Taxonomic Units) were performed using CD-HIT-OTU with a 97% identity cut-off [24]. Taxonomical assignment was performed against the Greengenes core database (version 13.8). OTUs were then rarefied to the lowest sequence count (n = 6641) in order to provide an equal depth of sequence analysis.

Sampling effort (Good’s coverage), rarefaction curve, and alpha diversity (Shannon and Simpson diversity indices, Chao1) were performed to obtain the community diversity. Beta-diversity analysis was used to compare the diversity between different treatments at different sampling periods. This includes the calculated Bray-Curtis distance and Weighed Unifrac distance plotted with Principal Coordinate Analysis (PCoA) to visualize the dissimilarity of microbial communities among different treatments at different periods. Raw sequences were deposited in the NCBI database (project accession number: SRP113663).

#### Real-time PCR assay

Extracted DNA was also subjected to quantitative real-time polymerase chain reaction (qPCR) to quantify the population of the selected microbes. The primers used are shown in Table 3.

**Table 3:** Primers used for qPCR reactions.

Targeted microbes	R/ F	Sequence 5′ to 3′	Product size (bp)	Reference
Total bacteria	R	CCATTGTAGCACGTGTGTAGCC	145	[25]
	F	CGGCAACGAGCGCAACCC		
Total protozoa	R	GCTTTCGWTGGTAGTGTATT	223	[26]
	F	CTTGCCCTCYAATCGTWCT		
Total fungi	R	CAAATTCACAAAGGGTAGGATGATT	121	[27]
	F	GAGGAAGTAAAAGTCGTAACAAGGTTTC		
Total methanogens	R	CGGTCTTGCCCAGCTCTTATTC	160	[28]
	F	CCGGAGATGGAACCTGAGAC		
Methanobacteriales	R	TACCGTCGTCCACTCCTT	343	[29]
	F	CGWAGGGAAGCTGTTAAGT		
*Fibrobacter succinogenes*	R	CGCCTGCCCCTGAACTATC	122	[27]
	F	GTTCGGAATTACTGGGCGTAAA		
*Ruminococus albus*	R	CCTCCTTGCGGTTAGAACA	175	[25]
	F	CCCTAA AAGCAGTCTTAGTTCG		
*Ruminococus flavefaciens*	R	CCTTTAAGACAGGAGTTTACAA	259	[25]
	F	TCTGGAAACGGATGGTA		
*Butyrivibrio fibrisolvens*	R	CCAACACCTAGTATTCATC	417	[30]
	F	GYGAAGAAGTATTTCGGTAT		

R: Reverse primer; F: forward primer; bp:base pair

Real-time quantitative PCR was performed for the extracted DNA using the BioRad CFX96 Touch (BioRad, USA). The qPCR reactions were performed in a total volume of 25 μL using the Maxima SYBR Green Mastermix (Thermo Scientific, USA). The reactions consisted of 12.5 μL SYBR Green Supermix, 1 μL of each reverse primer and forward primer, 2 μL of extracted DNA samples, and 8.5 μL of DNase-free H_2_O. The qPCR conditions were applied to all samples which consisted of an initial incubation at 94°C for 5 min, followed by 40 cycles of denaturation at 94°C for 20 s, annealing for 30 s, and extension at 72°C for 20 s. The annealing temperatures for primers of total bacteria, total fungi, total protozoa, total methanogens, *Butyrivibrio fibrisolvens*, *Ruminococcus albus and Fibrobacter succinogenes* were 55°C, while that for *Ruminococcus flavefaciens* and Methanobacteriales species were at 58°C [31].

### Statistical analysis

Since measurements were made on the same experimental unit (Goat), a repeated measures ANOVA with one experimental factor (Diets) and one repeated factor (Periods) has been considered. Animals were treated as genuine replications and Linear mixed effects models were utilized with both ‘Diets’ and ‘Periods’ as fixed effects and ‘Animals’ as random effect to capture appropriate structure for ANOVA. A full factorial model was considered with the two fixed effects. Pairwise comparisons of means between treatments were carried out using Fisher’s Least Significant Difference test. P-values <0.05 were considered statistically significant, P-values ≥0.05 and <0.1 were considered a trend. All analyses were carried out using the R software version 3.3.3 (R Core Team (2017)) (package nlme with function lme for carrying put linear mixed effects modeling). Since blood hematology and biochemistry analysis were conducted only at the end of the study (12-week period), the above two sets of data were analyzed as a one way ANOVA. For the metagenomics analyses, all assigned taxonomies were analyzed using Statistical Analysis of Metagenomic Profiles (STAMP) software [32], applying the Welch’s two-sided t-test for comparison among different sample groupings and produced P-values for statistical analysis within each period.

## Results

### Dry matter intake and digestibility

The fermentation of PKC to produce lovastatin for the feeding trial in this study adopted the protocol reported by Jahromi et al. [15]. The content of lovastatin in the fermented PKC differed among batches. However, the average lovastatin content in the treated (fermented) PKC used for this experiment was 850 mg lovastatin/kg DM (ranged 800–900 mg/kg) which contained 73.5% the active H-form (ranged 56–91%) while the remaining 26.5% were in the less active lactone form. The treated PKC from different batches were pooled together for each feeding period and their actual lovastatin concentration was used to calculate the different proportion of treated and untreated PKC to achieve the targeted levels of lovastatin for the different treatment groups.

Overall, dry matter intake (DMI) decreased with increasing level of lovastatin in the diet but only the DMI for the 6 mg/kg BW treatment was significantly lower than the control (Table 4). This observation was consistent over the three measuring periods. Apparent total-tract DM digestibility (DMD) was not affected by level of lovastatin, and averaged 57.2% for the control and 56.3% for the treatment groups. Period did not affect DMI and DMD and there were no diet x period effects on the DMI and DMD.

**Table 4:** Effects of increasing levels of lovastatin on dry matter intake (DMI, g/day), apparent total-tract dry matter digestibility (DMD, %) and digestible dry matter intake (DDMI, g/day) in goats.

	Dietary treatments				Period (weeks)				Significance		
	Control	Low	Medium	High	4	8	12	SEM	Diet	Period	Diet*Period
DMI	519.45^a^	512.64^a^	467.95^ab^	379.91^b^	441.31^y^	479.94^x^	488.72^x^	12.106	0.019	0.008	0.703
DMD	57.19	56.79	56.43	55.73	58.59	54.62	56.40	0.758	0.922	0.131	0.839
DDMI	277.63^a^	291.72^a^	240.73^a^	204.22^b^	253.58	263.42	275.77	6.854	0.003	0.051	0.597

Different superscript lowercase letters in each row within a treatment variable indicate significant differences

Control, Low, Medium and High represent 0, 2, 4 and 6 mg lovastatin/kg BW/day, respectively

### Methane emission

Mean CH_4_ emissions were 19.31 L/day for goats in the control group (0 lovastatin) and decreased steadily to 17.81, 14.95 and 11.80 L/day, respectively, for the Low, Medium and High treatments (Table 5). There was no significant difference in CH_4_ emission between the Low treatment group and the control. On the other hand, CH_4_ emissions from the Medium and High treatment groups were lower than the control group with an average reduction of 22.6% and 38.9%, respectively. When CH_4_ reduction was adjusted to a per kg DMI (recorded during the two days gas measurement period) basis, CH_4_ emission was 36.98 L/kg DMI for the control group and decreased (P<0.05) to 32.72 (7.8% reduction), 29.43 (20.4% reduction) and 29.21 L/kg DMI (21.0% reduction), respectively, for the Low, Medium and High treatment groups. There were period effects on CH_4_ emission (L/day) with the 8- and 12-week measurements being higher (P<0.001) than the first (4-week) measurement. However, no period effect was detected when CH_4_ emission was adjusted to a DMI basis.

**Table 5:** Effects of increasing levels of lovastatin on methane emission from goats.

	Dietary treatments				Period (weeks)				Significance		
	Control	Low	Medium	High	4	8	12	SEM	Diet	Period	Diet *Period
DMI (g/day)	522.26^a^	543.83^a^	504.92^a^	406.96^b^	460.71^y^	504.65^x^	500.34^x^	11.550	0.0141	0.0030	0.6394
CH4/day (L)	19.31^a^	17.81^ab^	14.95^bc^	11.80^c^	13.93^y^	17.12^x^	16.85^x^	0.564	0.0015	0.0001	0.5128
CH4/DMI (L/kg)	36.98^a^	32.72^b^	29.43^b^	29.21^b^	30.28	32.87	33.11	0.733	0.0029	0.0956	0.3320

Different superscript lowercase letters in each row within a treatment variable indicate significant differences

DMI: Dry matter intake; Control, Low, Medium and High represent 0, 2, 4 and 6 mg lovastatin/kg BW/day, respectively

### Microbial populations

A total of 752,869 high-quality sequence reads were generated from the rumens of 20 goats, sampled at three different periods (4 weeks, 8 weeks and 12 weeks). These sequences were assigned to 13,103 OTUs of archaea and bacteria based on a 97% similarity cutoff. In order to ensure an equal sequence depth were used for the downstream analyses, sequence number was normalized to the lowest number of sequence per sample (n = 6,641). Good’s coverage indices which approaches 100% indicates that the 16S rRNA sequencing libraries were able to represent the majority of the OTUs present in all of the samples sequenced.

Based on taxonomic assignment against the Greengenes database, 21 phyla were identified with *Bacteroidetes* (64.76 ± 7.90%) and *Firmicutes* (28.20 ± 7.51%) as the dominant phyla across all age groups in goat rumens (S1 Fig).

**Fig 1:**
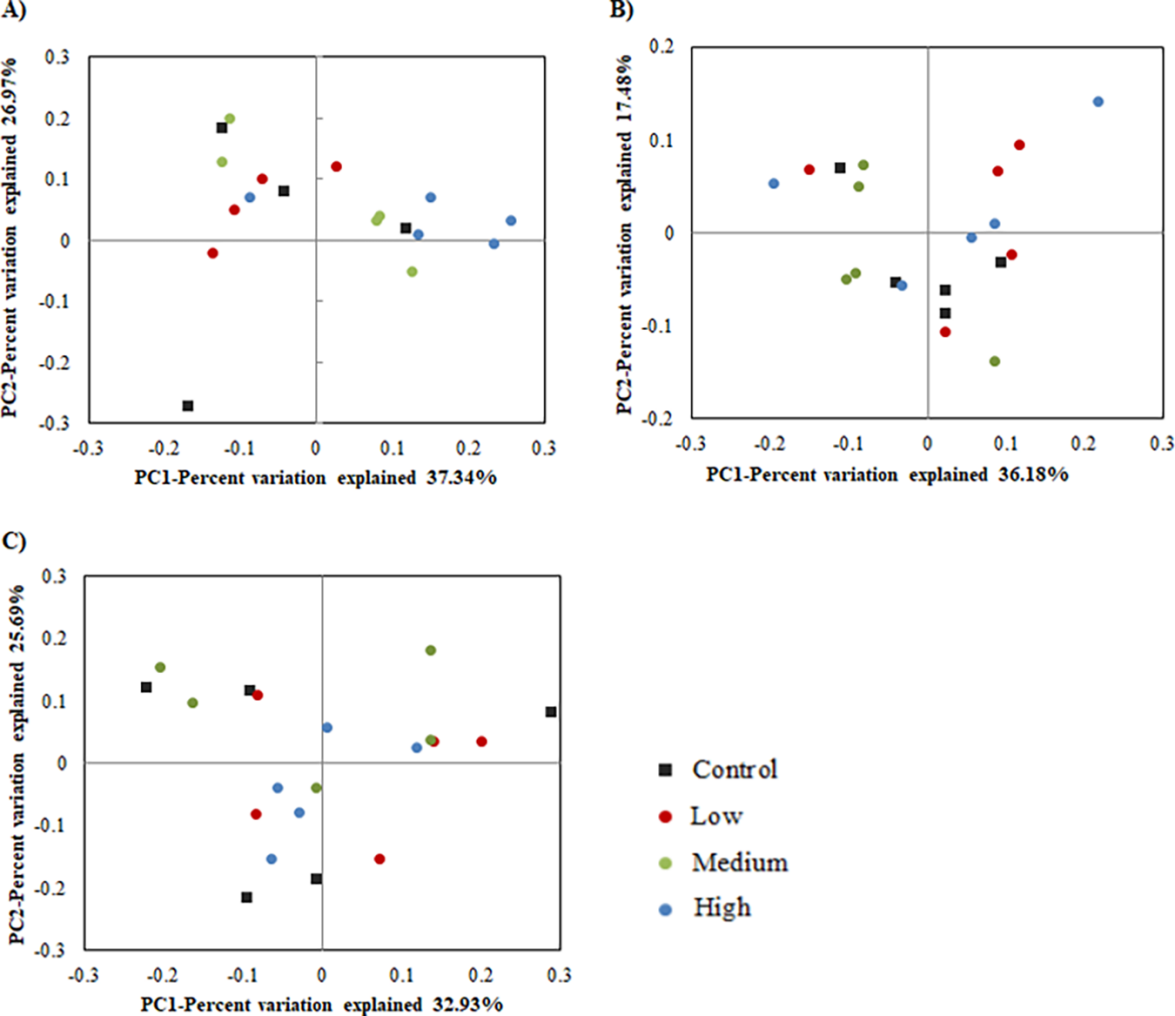
Principal coordinate analysis (PCoA) of taxonomic classification up to genus level using Bray-Curtis distance. A) 4 weeks period, B) 8 weeks period and C) 12 weeks period goats fed with different level of lovastatin.

The total bacterial diversity as measured using Chao1, Shannon and Simpson indices (Table 6) showed no noticeable differences in species richness with increased level of lovastatin and with period effect. Beta diversity was calculated using Bray-Curtis distances and visualized by PCoA (Fig 1 and S2 Fig). In general, no clear clustering patterns based on period were observed (S2 Fig). However, the communities from week 4 and week 8 were more closely related to each other than to the week 12 community. In addition, a treatment dependent clustering of rumen microbiota was observed in the first period, whilst those of control groups show differences, implying a more varied flora (Fig 1).

**Table 6:** Number of OTUs and diversity indices from rumen of goats fed with increasing levels of lovastatin.

Period (weeks)	Dietary treatments	OTUs	Chao1	Shannon	Simpson	Good’s Coverage
4	Control	218.8 ± 18.46	260.59 ± 25.13	5.15 ± 0.31	0.93 ± 0.03	99.64 ± 99.68
	Low	214.4 ± 18.9	247.52 ± 19.52	5.18 ± 0.5	0.93 ± 0.04	99.62 ± 99.56
	Medium	217 ± 21.41	250.75 ± 23.14	5.31 ± 0.4	0.94 ± 0.02	99.6 ± 99.65
	High	210.4 ± 31.92	244.32 ± 24.27	4.84 ± 0.71	0.91 ± 0.05	99.67 ± 99.67
8	Control	212.4 ± 20.6	240.47 ± 29.99	5.31 ± 0.32	0.95 ± 0.02	99.66 ± 99.66
	Low	213.6 ± 16.74	249.48 ± 18.8	5.11 ± 0.39	0.93 ± 0.03	99.65 ± 99.65
	Medium	243.8 ± 6.98	272.67 ± 13.81	5.37 ± 0.28	0.95 ± 0.02	99.76 ± 99.69
	High	210.6 ± 17.73	248.66 ± 28.12	5.27 ± 0.41	0.94 ± 0.03	99.6 ± 99.64
12	Control	221.2 ± 12.15	262.43 ± 21.09	5.2 ± 0.4	0.93 ± 0.03	99.65 ± 99.65
	Low	212 ± 10.7	249.94 ± 11.95	5.38 ± 0.43	0.94 ± 0.03	99.62 ± 99.63
	Medium	231.6 ± 27.17	270.89 ± 26.21	4.88 ± 0.6	0.91 ± 0.04	99.7 ± 99.69
	High	214.8 ± 20.67	254.06 ± 26.65	5.51 ± 0.25	0.95 ± 0.01	99.46 ± 99.41

Control, Low, Medium and High represent 0, 2, 4 and 6 mg lovastatin/kg BW/day, respectively

The effects of different doses of lovastatin on the population of *Archaea* and other selected microbes quantified by qPCR are shown in Table 7. Surprisingly, results of this study showed that lovastatin had small effects on the rumen microbial populations. Protozoa were decreased at Low treatment group but were similar for Medium and High treatment groups. *Ruminococus flavefaciens* decreased with increasing levels of lovastatin and the High treatment group was significantly different from the control. None of the other bacteria examined were affected by the increasing levels of lovastatin. Methanogens and Methanobacteriales only tended to be lower in the treatment groups compared to the control group.

**Table 7:** Effects of increasing levels of lovastatin on microbial population log10 copy number/ml rumen fluid.

	Dietary treatments				Period (weeks)				Significance		
	Control	Low	Medium	High	4	8	12	SEM	Diet	Period	Diet*Period
*Butyrivibrio fibrisolvens*	4.88	4.91	4.76	4.73	4.68^y^	4.82^xy^	4.96^x^	0.036	0.228	0.008	0.807
*Ruminococus albus*	6.61	6.04	6.25	6.13	6.46	6.30	6.01	0.090	0.147	0.123	0.736
*Fibrobacter succinogenes*	5.86	5.94	5.91	5.98	5.66^y^	5.88^y^	6.22^x^	0.075	0.968	0.002	0.274
*Ruminococus flavefaciens*	4.83^a^	4.78^a^	4.61^a^	3.68^b^	4.23	4.74	4.45	0.120	0.004	0.158	0.410
Total fungi	5.77	5.42	6.08	5.88	5.78	5.91	5.67	0.097	0.343	0.368	0.043
Total protozoa	4.82^a^	3.68^b^	4.53^a^	4.80^a^	3.97^y^	4.01^y^	5.39^x^	0.186	0.032	<0.001	<0.001
Total bacteria	8.42	8.60	8.46	8.41	8.36^y^	8.54^x^	8.51^x^	0.031	0.205	0.013	0.068
Total methanogen	5.60	5.14	5.38	5.21	5.03^y^	5.22^y^	5.75^x^	0.087	0.223	0.002	0.835
Methanobacteriales	4.07	3.96	3.98	3.85	3.46^y^	3.68^y^	4.75^x^	0.103	0.807	<0.001	0.001

Control, Low, Medium and High represent 0, 2, 4 and 6 mg lovastatin/kg BW/day, respectively

Different superscript lowercase letters in each row within a treatment variable indicate significant differences

Over time the total number of bacteria increased, as did *Fibrobacter succinogenes* and *Butyrivibrio fibrisolvens*. Along with the bacteria, the number of protozoa as well as the total methanogens and the Methanobacteriales increased from 4 to 12 weeks.

### Volatile fatty acids profile and pH

The effects of different dosage of lovastatin on pH and VFA content of rumen fluid are presented in Table 8. There was no significant difference in acetate concentration, however, increased propionate concentration was observed with increasing level of lovastatin dosage, especially for the Medium and High treatment groups, which resulted in a lower A/P ratio, as compared to the control. Total VFA production increased with increasing dosage of lovastatin but only the Low lovastatin group was significantly higher than the control. On the other hand, the isobutyrate concentration in the High lovastatin group was lower than the control and the other treatment groups. There was a period effect on most of the VFAs measured. The effects are inconsistent with acetate and propionate, and particularly the total VFA increased over time of measurement (e.g. 4-, 8- and 12-week periods), while others (isopropionate and isobutyrate) decreased.

**Table 8:** Effects of increasing levels of lovastatin on rumen pH and VFA content.

	Dietary treatments				Period (weeks)				Significance		
	Control	Low	Medium	High	4	8	12	SEM	Diet	Period	Diet*Period
pH	6.62	6.59	6.76	6.73	7.02^x^	6.97^x^	6.05^y^	0.066	0.422	<0.001	0.877
Acetic (A, %)	54.57	54.97	53.88	55.33	53.17^y^	54.45^y^	56.43^x^	0.351	0.631	0.001	0.961
Propionic (P, %)	21.76^b^	22.72^ab^	23.01^a^	23.64^a^	22.94^x^	23.36^x^	22.05^y^	0.203	0.010	0.012	0.185
Isopropionic (%)	2.33	2.25	2.37	2.10	2.79^x^	2.35^y^	1.65^z^	0.078	0.383	<0.001	0.694
Butyric (%)	16.29	15.02	15.75	14.77	15.47	14.84	16.07	0.263	0.405	0.0734	0.558
Isobutyric (%)	5.05^a^	5.03^a^	5.00^a^	4.17^b^	5.63^x^	5.00^y^	3.80^z^	0.158	0.050	< .001	0.580
Total (mM/mL)	74.24^b^	78.63^a^	73.68^b^	76.10^ab^	66.34_z_	69.68^y^	90.97^x^	1.577	0.062	< .001	0.183
A/P	2.54^a^	2.43^ab^	2.35^b^	2.35^b^	2.33^y^	2.34_y_	2.58^x^	0.031	0.080	0.002	0.169

Control, Low, Medium and High represent 0, 2, 4 and 6 mg lovastatin/kg BW/day, respectively

Different superscript lowercase letters in each row within a treatment variable indicate significant differences

### Blood parameters

Data from the blood biochemistry and hematology analyses are useful indicators for any physiological stress on the animals. The results showed no significant differences in most of the parameters measured, except for mean corpuscular volume (MCV), eosinophils, basophils, and also the total cholesterol, high- density lipoproteins (HDL) and low-density lipoproteins (LDL) (Table 9). MCV was lowest in the Low treatment compared to the other treatments, while eosinophil and basophil cells were lower in all lovastatin-treated groups, compared to the control. Supplementation of lovastatin, including the Low treatment significantly lowered the total cholesterol, LDL and HDL (P<0.001) concentrations as compared to the control group.

**Table 9:** Effects of increasing levels of lovastatin on blood biochemistry and hematology.

	Dietary treatments				SEM	Significance
	Control	Low	Medium	High		
Enzymes (U/L)						
Alkaline phosphatase	184.20	356.20	141.80	250.00	59.459	0.639
Aspartate aminotransferase	72.20	67.40	71.60	140.00	15.736	0.314
Alanine aminotransferase	27.60	23.00	25.60	21.40	1.903	0.702
Lactate dehydrogenase	218.60	194.00	202.80	252.40	16.576	0.648
Creatine kinase	160.80	239.20	135.00	272.20	26.627	0.226
Cholesterol (mmol/L)						
Triglyceride	0.37	0.31	0.34	0.33	0.021	0.784
Cholesterol	1.86^a^	0.94^b^	0.72^b^	0.64^b^	0.120	<0 .001
Low density lipoprotein	0.62^a^	0.31^b^	0.31^b^	0.37^b^	0.036	0.001
High density lipoprotein	1.43^a^	0.88^b^	0.67^c^	0.71^bc^	0.075	<0 .001
White blood cells (x10^9^/L)						
Total white blood cells	12.48	10.91	9.44	9.28	0.665	0.300
Neutrophils	6.52	6.15	5.38	4.52	0.356	0.195
Lymphocytes	3.12	3.20	2.89	3.59	0.276	0.864
Monocytes	0.63	0.64	0.49	0.51	0.040	0.459
Eosinophils	1.71^a^	0.59^b^	0.35^b^	0.45^b^	0.185	0.017
Basophils	0.30^a^	0.14^b^	0.16^b^	0.12^b^	0.026	0.040

Control, Low, Medium and High represent 0, 2, 4 and 6 mg lovastatin/kg BW/day, respectively

Different superscript lowercase letters in each row indicate significant differences

### Residues in tissues

The concentrations of lovastatin residue in all tissues (LD muscle, brain, liver and kidney) for all treatments were below the detectable levels by the HPLC procedure. In addition, results from the LCMS analysis for samples of each type of tissues of the highest level (i.e. 6 mg/kg BW) also showed very low lovastatin concentrations of between 0.01 to 0.03 μg/ g of tissue.

## Discussion

Enteric CH_4_ emission is a natural phenomenon occurring during the anaerobic digestion in animals, particularly in ruminants. Although significant research efforts have been invested over the last two to three decades with the goal of reducing emissions of this potent greenhouse gas from ruminant livestock, no single CH_4_ mitigation approach has been widely accepted by farmers. This is because the effect has been either short-term or the mitigating agents used to suppress activity of the methanogens have also adversely affected other useful bacteria in the rumen ecosystem and ultimately animal production. The above shortcomings have led to the search of specific compounds, such as statins, a class of HMG-CoA reductase inhibitors which specifically interfere with the biosynthesis of cell membranes of archaea, and thus selectively suppressing growth of methanogens. However, there are still several unclear issues in the use of these compounds including: the optimal dosage and long term effects of statins to effectively mitigate enteric CH_4_ emission; their effect on health and wellbeing of the host animals; and the possible accumulation of the statins in the edible tissues and milk of the animals.

Results of our 12 week study showed no adverse physiological or behavioral effects except that some animals in the higher dosage groups (particularly the 6 mg /kg BW) did not consume all the feed offered to them. Our results also showed that the low feed intake of the higher lovastatin supplemented animals persisted over the whole experiment. It is not clear whether the lower DMI in the higher lovastatin treatment was due to poor palatability of the fermented PKC or a direct effect of lovastatin on the rumen ecosystem and host. The fact that lovastatin supplementation has not significantly affected the overall microbial (including the methanogens) populations in the rumen (Table 7) and did not impact the apparent total-tract DM digestibility coefficients among the treatments seems to suggest that palatability of the feed could be the main influencing factor for the low DMI. However, the above speculation requires further investigation.

Enteric CH_4_ emission is dependent on several factors, including quantity and quality of feed, and has been shown to be highly correlated to DM intake [33–35]. The DMI of the goats in the High treatment group was lower (P<0.05) than the Control, Low and Medium groups. Therefore, the CH_4_ emissions among treatments were also compared based on DMI (L CH_4_/kg DMI) to eliminate the effect of differential DMI among treatments on CH_4_ emission. Results of our study showed that lovastatin at Low dosage (2 mg lovastatin/kg BW) reduced CH_4_ emission per kg DMI by 11.4%, while the reduction increased to about 21% for the Medium and High dosage levels (4 and 6 mg/kg BW, respectively), as compared to the control. Since there was a reduction in NDF content of the lovastatin-treated diets compared to the control (Table 2), the lower CH_4_ emission of goats in the lovastatin-treated (particularly the Medium and High treatment groups) could also partially be an effect of lower NDF intake. We do not totally rule out this possibility but would argue that NDF intake is not the primary factor affecting CH_4_ emission in this study. This is because the NDF intake of goats in the High treatment group was 18.86% lower than that of goats in the Medium treatment group (182.73 vs. 225.19 g/ day) but there was no further reduction in CH_4_ emission from goats fed with the High dosing level (lower NDF intake) as compared with those fed in the Medium treatment (higher NDF intake) group. In addition, results of regression analysis on effects of level of feeding and diet quality on CH_4_ emission in ewes showed that the strongest relationship found was between DMI and CH_4_ emission where DMI alone explained for 91% of the variation in CH_4_ emission. Including feed quality variable (e.g. lipid or NDF) in the relationship only improved the prediction equation marginally with the r^2^ increasing from 0.91 to 0.94 [36].

The results from this study suggest that the optimal dosage of naturally produced lovastatin to mitigate enteric CH_4_ emission in goats would be around 4 mg/kg BW. No further reduction in methane emission with dosage higher than 4 mg/kg BW was observed. The suggested optimal dosage from the present study is close to the proposed 4.34 mg Lovastatin/kg BW from red yeast rice used by Wang et al. [12] in goats. However, the authors obtained only 13% reduction in CH_4_ emission while the reduction achieved with the naturally produced lovastatin in our experiment was 20.4%. Morgavi et al. [37] supplemented red yeast rice (2.26 mg lovastatin /kg BW) to sheep for 2 weeks reported a 30% reduction in enteric CH_4_ emission as early as on the third day of feeding. However, these differences in methane emissions could be due to diet effects and/or differences in the rumen microbial community. On the other hand, feeding fermented rice containing 2.2 mg lovastatin /kg BW, even only for four days, resulted in a decreased feed intake and critical clinical manifestations of digestive, muscular and urinary system disorders in cattle [11]. Data from the literature and the current study suggest that cattle are less tolerant towards lovastatin as compared to sheep and goats, while variability in the rate of CH_4_ mitigation within species could be due to differences in the concentrations of the active hydroxyacid form in the compound as demonstrated by Faseleh Jahromi et al. [38]. The concentration of the active hydroxyacid lovastatin in the red yeast rice used by Wang et al. [12] was 56% while in this study it, averaged 73.5%. Importantly, the effect of lovastatin on CH_4_ emission of goats in this study was similar during all three measurement periods, suggesting that the dietary supplementation of lovastatin to mitigate CH_4_ emission can be effective for at least 12 weeks.

Based on the metagenomics study, lovastatin did not influence the rumen microbial diversity or richness. In this study, *Bacteroidetes* and *Firmicutes* were the dominant phyla detected in the rumen fluid of goat, a result in accordance to those of Wang et al. [21] and Henderson et al. [39]. Being a HMG-CoA reductase inhibitor, lovastatin should inhibit the biosynthesis of the cell membranes of archaea and suppress growth of methanogens as reported in several studies [10, 12, 40]. Surprisingly, our qPCR results did not concur with the above. In our study, total methanogen and Methanobacteriales species (known to be responsible for most CH_4_ production) of the lovastatin treatment groups tended to be lower but were not statistically significant compared to the control (P>0.05). Although the study of Wang et al. [12] reported a lower CH_4_ reduction as compared to the present study (13 vs. 20.4%), they observed a significant reduction in the proportion of *Methanobrevibacter*. However, the abundance of some archaea remained unchanged or even increased (such as *Methanomicrobium*) by lovastatin supplementation. The above authors suggested that possibly some but not all the archaea need HMG-CoA reductase in their growth and proliferation. Our study showed that total methanogen and methanobacteriales, and several others (total protozoa, total bacteria, *Fibrobacter succinogenes* and *Butyrivibrio fibrisolvens*) increased with time, especially in the 12-week measurement compared to the 4-week measurement (P<0.01). The increase could be due to the differences in the rumen fluid sampling protocols (oral stomach tube in 4- and 8-week against direct collection from rumen of slaughtered the animals for 12-week samples).

The effect of lovastatin supplementation on rumen fermentation characteristics was also investigated in this study to provide further understanding on the effects of lovastatin on the enteric CH_4_ emission process. Total VFA production was not affected by lovastatin supplementation which concurred with the fact that lovastatin had little influence on rumen microbial populations and also did not affect diet digestibility. The effect of lovastatin on VFA profiles was not very consistent except that increasing dosage of lovastatin (Medium and High treatment groups) increased the proportion of propionate as compared to the control (P<0.01), while the proportion of acetate was not different (P>0.5) among treatments. The positive effect of lovastatin on propionate production in this study suggests that dietary supplementation of lovastatin modifies the fermentation pathway to favor propionate production in the rumen, which served as an alternative H_2_ sink, competing with methanogens for CH_4_ production. In addition, the decrease in *Ruminococcus* as observed in this study would lead to a decreased acetate production and consequently a lower production of H_2_ available for methane production. This finding also agrees with Azlan et al. [13] which reported increased in propionate in goats offered lovastatin; and Faseleh Jahromi et al. [10] which also found a higher proportion of propionate during an *in vitro* incubation with fermented rice straw compared to the control. There was a period effect on most of the VFAs measured. The effects were inconsistent with the different VFAs but total VFA production of the 12-week increased nearly 1.5-fold as compared to the 4-week measurement. The above, together with the higher rumen microbial population for the 12-week sampling could be due to differences in rumen fluid sampling protocols suggested earlier.

Long-term consumption of statin to treat hyperlipidemia can result in myotoxicity of skeletal muscle in humans [41–42]. To the best of our knowledge, no data on myotoxicity due to long-term consumption of statins in farm animals has been reported. However, dietary supplementation of red yeast rice containing lovastatin up to 2.6 mg/kg BW showed adverse digestive and other physiological disorders in cattle [11]. Thus, overdose administration of this compound in livestock production could adversely affect the health and wellbeing of the animals leading to negative animal welfare issues. The side effects on skeletal muscle associated with the use of statins are dose-dependent [43], therefore, a safe level of feeding the compound to the animals must be determined. Our results showed no detectable lovastatin in all the tissues analyzed using HPLC while the LCMS/MS analysis of the samples from the High lovastatin treatment group fed for 12 weeks recorded only between 0.01 to 0.03 μg/ g (10–30 μg/kg) lovastatin in tissues. Assuming that someone was to eat 200 g of meat containing the above residue of lovastatin, he would have consumed only 6 micrograms of statin, which is far less than the lowest daily dose (10 mg/ day) recommended for treatment of hypercholesterol in humans [44].

Blood biochemistry and hematology data play a vital role in indicating the physiological, nutritional and pathological status of the animal [45]. Blood alkaline phosphatase (ALP), aspartate aminotransferase (AST) and alanine aminotransferase (ALT) are useful indicators of the function, integrity and infection in the heart and liver [46–47]. The non-significant difference in these three enzymes together with blood creatine kinase (CK) level, which is associated with inflammation and damage in muscle cell and tissues [48–49], between the control and treatment groups showed that supplementation of lovastatin in the High treatment group (6 mg/kg BW) did not bear negative impact or toxicity towards the animal. Basophils and eosinophils belong to the white blood cells group and are components of allergic inflammation. In this study, the supplementation of increasing levels of lovastatin reduced the counts of basophils and eosinophils. In fact, the eosinophils count for all treatment groups, and the basophils count for the High treatment group were within the reported normal range of 0.05–0.65 x10^9^/L and 0–0.12 x10^9^/L for eosinophils and basophils, respectively [50], while that of control was not. Histamine released by basophils is modulated by histaminases of eosinophils, in response to parasitic infections or inflammation of the gastrointestinal or respiratory tract [51]. Therefore, our results indicated that the animals fed with lovastatin were actually healthier in several respects.

Results of the present study show that lovastatin produced from fermentation of PKC with *Aspergillus terreus* was effective in reducing the blood total cholesterol, LDL and HDL (P<0.001). The mode of action of statins is that they inhibit the rate-limiting enzyme of cholesterol synthesis in the liver, leading to lower production of LDL [52]. Vaughan et al. [52] also reported that an upregulation of expression of hepatic LDL receptors, which then lowered the concentrations of LDL in blood. In agreement with Wang et al. [12] and Azlan et al. [13], since statin is a medication known in human to treat hyperlipidemia, this study also reported a reduction in blood cholesterol in goats that consumed a diet containing lovastatin. This could be attractive to the producer to produce meat with a lower cholesterol content.

The following conclusions can be drawn from the results of this study:

1. Naturally produced lovastatin using PKC as substrate can effectively decrease enteric methane emission in goats. The optimal dosage level is 4 mg/kg BW which reduced CH_4_ /kg DMI by about 20% compared to the control (no lovastatin). No further reduction in CH_4_ emissions would be expected with a higher dosage of lovastatin.
2. There was no significant difference in CH_4_ emission per kg DMI over the three periods indicating the long-term effectiveness (at least 12 weeks) of the *in situ* produced lovastatin from PKC in mitigating enteric CH_4_ emission.
3. The minimal effects of lovastatin on the population of rumen microbes including total methanogen and Methanobacteriales (known to be responsible for CH_4_ production) is rather unexpected, since significant reduction in CH_4_ emission was observed for goats in the Medium and High treatment groups. However, the supplementation of increasing levels of lovastatin increased the production of propionic acid, suggesting a shift in metabolic hydrogen sink from CH_4_ production. Thus, this could partially explain for the reduction in CH_4_ emission in goats from the Medium and High treatment groups.
4. No adverse physiological effects were noted in the experimental animals except that lovastatin (or the treated PKC) can have a negative effect on the palatability of the formulated diet.
5. The amount of lovastatin residue detected in meat, liver, kidney and brain samples of goats from the High treatment group was 0.01–0.03 μg/g and therefore, the amount of residue is far below the level recommended for hyper-cholesterol treatment in human.

## Supporting information

S1 Fig The composition of all microbiome at phylum level of goat’s rumen. (P1, P2, P3 = period and C, L, M, H = 0, 2,4 and 6 mg/kg BW).
(TIF)

S2 Fig Principal coordinate analysis (PCoA) of the community structure using Bray-curtis distances. The three pictures show the principle coordinates (PC1, PC2, and PC3) from different angles. Red triangle indicates week 4, blue square indicates week 8 and orange dots represent week 12.
(TIF)

S1 File Minimal data set. (XLSX)
