# Supplementary figures and images for: Effects of naturally-produced lovastatin on feed digestibility, rumen fermentation, microbiota and methane emissions in goats over a 12-week treatment period
Source: PLoS One. 2018 Jul 5;13(7):e0199840. doi: 10.1371/journal.pone.0199840 (PMC6033401; doi:10.1371/journal.pone.0199840)

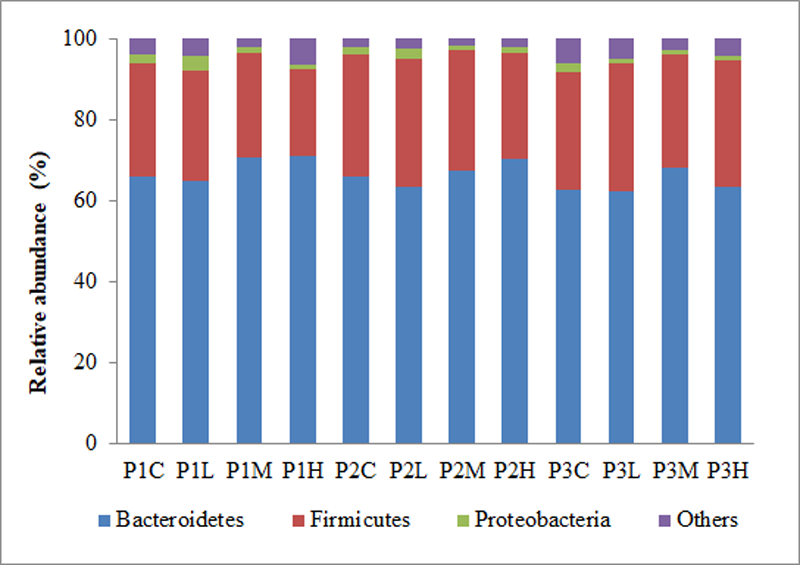

Supplement: S1 Fig — (P1, P2, P3 = period and C, L, M, H = 0, 2,4 and 6 mg/kg BW). (TIF) [file pone.0199840.s001.tif]

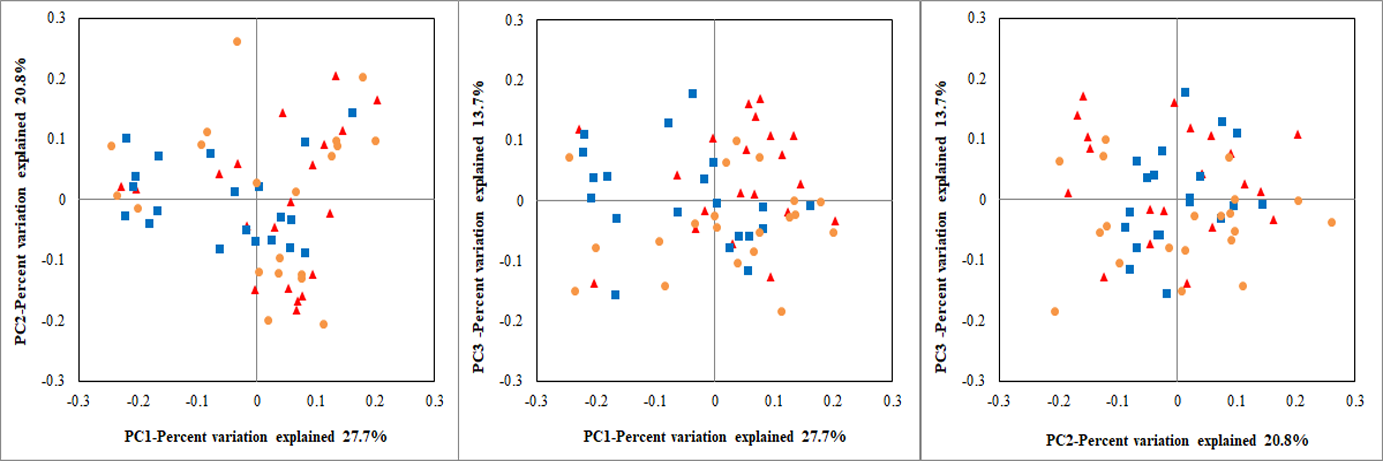

Supplement: S2 Fig — The three pictures show the principle coordinates (PC1, PC2, and PC3) from different angles. Red triangle indicates week 4, blue square indicates week 8 and orange dots represent week 12. (TIF) [file pone.0199840.s002.tif]
